# Electronic Nicotine Delivery Systems and Serum Folate: A Case Study

**DOI:** 10.1177/1179173X19885397

**Published:** 2019-11-20

**Authors:** Julie M Croff, Ashleigh L Chiaf, Micah L Hartwell, Erica K Crockett, Chibing Tan, Kent Teague

**Affiliations:** 1 Center for Wellness & Recovery, Oklahoma State University Center for Health Sciences, Tulsa, OK, USA; 2 Center for Rural Health, Oklahoma State University Center for Health Sciences, Tulsa, OK, USA; 3 Master of Public Health Program, Graduate College, Oklahoma State University, Stillwater, OK, USA; 4 School of Community Health Sciences, Counseling and Counseling Psychology, College of Education, Health and Aviation, Oklahoma State University, Stillwater, OK, USA; 5 Integrative Immunology Center, Department of Surgery, The University of Oklahoma, Tulsa, OK, USA

**Keywords:** ENDs, electronic cigarettes, folate, folic acid

## Abstract

**Introduction:**

In 2018, the US Surgeon General declared youth electronic nicotine delivery systems (ENDS) use as an epidemic. Combustible cigarettes have been shown to adversely affect folate status; however, no study to date has explored how ENDS impact folate status.

**Methods:**

In this case study, a white 18-year-old woman was followed for a 1-month period as part of a larger study. During her participation in the study, a self-report of dietary folate consumption and use of alcohol, tobacco, and other drugs (ATOD) was collected. Each week, dried blood spots were collected to assess red blood cell (RBC) and serum folate.

**Results:**

During the first 2 weeks of the study, she used ENDS and her serum folate values were depleted in the ranges of 1.91 to 4.39 µg/L. During the third week, when no ENDS were used, her serum folate value was measured at 29.44 µg/L. When ENDS use resumed during the fourth week, her serum folate value fell to 7.50 µg/L.

**Conclusions:**

This relationship suggests the need for additional studies on ENDS use and serum folate status, particularly among adolescent women who already have the lowest folate status nationally.

## Introduction

Prevalence of adolescent use of electronic nicotine delivery systems (ENDS) has reached an all-time high: the number of high school seniors reporting ENDS use doubled from 2017 to 2018 from 10.9% to 20.9%.^1^ This rapid increase in ENDS use resulted in the declaration of an epidemic by the US Surgeon General in 2018. Prevalence of adolescent ENDS use previously surpassed combustible cigarette use and remains the most commonly used tobacco product among the adolescent population.^2,3^ Early reports of ENDS use among adolescents suggested combined use with combustible cigarettes.^4^ However, the most recent literature suggests that higher proportions of adolescents use ENDS without combustible cigarettes.^5^ The prevalence of the use of these products may be a serious public health concern as very little is understood about how ENDS use may disrupt biological systems and, therefore, systematically affect the health of users.

Although little is known about the use of ENDS and nutritional status, repeated exposure of combustible cigarette smoke to bodily tissues has been shown to impact nutrient status,^6^ including the status of folate.^7^ Although there are many toxic compounds in combustible cigarette smoke, populations of free radicals may negatively impact folate status.^8^ Moreover, the free radicals found in inhalable aerosols of ENDS may have a similar effect.^9^ National studies have demonstrated that red blood cell (RBC) folate levels are lowest among 12- to 19-year-old adolescents,^10^ thereby potentially placing them at additional risk when combined with tobacco exposure. Folate is a critical nutrient involved in DNA synthesis, DNA methylation, and the production of multiple neurotransmitters through the biopterin cycle (eg, serotonin, dopamine, noradrenaline, and adrenaline).^11^ Related to these roles, folate deficiency is associated with megaloblastic anemia and depression.^11^ Depleted folate, at levels that may not meet a deficiency threshold, may still result in symptoms associated with anemia and depression, like lethargy.^12^ Moreover, among women of childbearing potential, folate status in the months before pregnancy is a key indicator of neural tube defects (NTDs) during pregnancy.^13^ To that end, women of childbearing potential are more likely to have folate status below the recommended threshold if they are current smokers.^13^

Mechanisms for the relationship between cigarette use and nutrient status fall under 2 categories: behavioral and physiological. Behavioral mechanisms are hypothesized to remain consistent across 2 categories of tobacco users: ENDS users and combustible cigarette users may consume less-nutrient-dense diets.^14,15^ Behaviorally, teenagers have among the lowest folate status in the United States and are most likely to use ENDS.^2,10^ Physiologically, however, there may be differences between ENDS and combustible cigarettes. Overall, exposure to tobacco smoke has been shown to decrease levels of serum folate.^7^ The critical unanswered question in the literature is whether ENDS elicit the same physiological depletion of folate as combustible cigarettes. The purpose of this case study is to describe an improvement in serum folate status that appears to be related to temporary termination of ENDS use.

## Methods

The following is a single case of a white 18-year-old woman who reported ENDS use at the time of enrolling in a larger study. The study from which this case study was collected measures nicotine and ENDS use as a covariate of the relationship between alcohol use and folate status. This research involving human subjects was approved by the Oklahoma State University Institutional Review Board (Application Number ED14115). The participant whose information is included in this case study provided her written informed consent for patient information to be published.

The participant consented for her data to be used as a case study and to participate in a 1-month, longitudinal research study of her substance use and dietary behaviors. Recruitment for this study employed respondent-driven sampling. Upon referral to the research staff, potential participants were directed to the study website (www.TulsaFABstudy.com), which includes a video explaining the study, frequently asked questions, and a link to a screening survey. The participant whose data are presented in this case study met the following inclusion criteria for selection into the 1-month study: female, between the ages of 14 and 24, lived within a 45-minute drive of the study office, and consumed at least 4 alcoholic drinks on 1 occasion during the past month prior to enrollment. Only female participants were recruited because this is part of a larger trial examining the periconceptional behaviors of women of childbearing potential. If a potential participant met screening criteria, a member of the lab called to re-screen and schedule a consent and baseline appointment. Participants under the age of 18 brought at least 1 parent to the initial appointment for informed consent.

At her baseline appointment, the participant completed a lengthy questionnaire of behavioral, environmental, and social covariates. After baseline, the participant would return weekly to the lab to give a self-report of daily alcohol, tobacco, ENDS, and other drug use. Finally, at each weekly appointment, she gave a self-report of past week folate consumption and a dried blood spot sample. The participant’s data were selected for this case study based on the following information: daily use of ENDS over a majority of the study period, self-report of the strength of e-liquid used in the ENDS product, abrupt abstinence of daily use of ENDS during the third week of the study period, and the variability of serum folate status in relation to ENDS use.

### Alcohol, tobacco, and other drug use

Use of alcohol, tobacco, and other drugs was assessed via self-report at baseline for the previous month’s use. Each week, the participant engaged in a timeline follow-back (TLFB) protocol of her use of alcohol, cigarettes, ENDS, and other drugs over the previous week.

### Folate consumption

Folate consumption over the previous week was assessed using the Block dietary folate equivalents (DFEs) screener, a 21-question screener wherein 19 questions ask about diet and 2 questions about supplement use. The participant completed the Block DFE screener at baseline and weekly thereafter throughout the study. The Block DFE screener has been validated against food frequency questionnaires, finding a low to moderate, but highly significant correlation between DFE consumption and RBC folate status.^16^

### Dried blood spots

Dried blood spots were collected on Arrayit blood cards, which use linear flow chromatography to separate whole blood serum from erythrocytes and leukocytes. A total of 5 Arrayit cards were collected from the participant: 1 at baseline and weekly thereafter over an approximate 28-day observation period. Erythrocyte folate values were assayed only at baseline as the values are a moving average of past 120-day consumption and absorption. They are not expected to vary greatly over a 28-day period. Serum folate values were assayed for each sample, as serum folate includes past 24-hour consumption and absorption. *Lactobacillus casei* microbiological assay of folic acid derivatives was completed with microtiter plates.

## Results

The following is a single case of a white 18-year-old woman who used ENDS with a strength of 3 mg/mL over the course of a 1-month period. During the first 2 weeks of the study, she used ENDS at 60 hits per day. However, she stopped ENDS use completely throughout the entirety of week 3. During week 4, she resumed use of ENDS at 60 hits per day. The baseline erythrocyte folate value was 227.16 ng/L, below the threshold of 400 ng/L set by the World Health Organization for women of childbearing potential. The relationship between folate consumption as well as serum folate level by ENDS use is shown in Table 1.

**Table 1.** Folate consumption and serum folate by ENDS use.

	Daily folate equivalents consumed during previous week (mcg DFEs)	Serum folate (µg/L)	ENDS frequency (hits/d)	ENDS strength (mg)	Self-reported drinking events (n)
Baseline	457.52	2.62	60	3	0
Week 1	100.63	1.91	60	3	1
Week 2	342.51	4.39	60	3	0
Week 3	100.76	29.44	0	0	0
Week 4	73.83	7.50	60	3	0

Abbreviations: DFE, dietary folate equivalent; ENDS, electronic nicotine delivery systems.

The consumption of DFEs varied widely across the study period (73.83-457.52 mcg DFEs per day). Two distinct ranges of consumption were observed: weeks 1, 3, and 4 were well below expected consumption, whereas consumption at baseline exceeded the threshold of 400 mcg DFEs per day for women of childbearing potential and week 2 approached the threshold. Serum folate values did not appear to co-vary with consumption.

**Figure 1.**
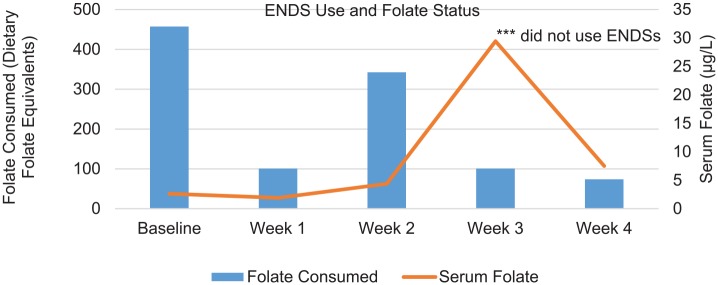
ENDS use, folate status, and folate consumption by week. ENDS indicates electronic nicotine delivery systems.

Serum folate values remained depleted while the participant regularly engaged in ENDS use over the first 2 weeks (1.91-4.39 µg/L). The participant reported engaging in 1 drinking event during week 2 of the study, the day after the blood sample was drawn for the week 2 folate and 6 days prior to the blood sample for week 3. The participant reported that this drinking event consisted of consuming 12 drinks over a 2-hour period. During week 3, the serum folate value of the participant significantly increased without daily ENDS use. Notably, DFE consumption was approximately the same in weeks 1 and 3. The only notable difference in week 3 was her ENDS use (see Figure 1). Finally, in week 4, after resuming ENDS use, her serum folate value was much lower than the previous week.

## Discussion

The data presented on the relationship between serum folate status and ENDS use provide insight for further research. In addition, this finding may be important among women of childbearing potential, particularly those in the youngest age cohort (12-19) who have the lowest folate status in the nation.^10^ ENDS use may operate through a similar physiological pathway as combustible cigarettes resulting in depletion of folate status, in spite of behavioral folate consumption. If this pattern is identified more broadly, prevention approaches to increase folate status among ENDS users should be tested. Thus, specific emphasis for prevention approaches should be among young women of childbearing potential as nicotine exposure remains associated with NTDs.^17^

Additional examination of ENDS use and folate status is warranted. Adolescents perceive low levels of risk associated with ENDS use.^5^ However, it is unknown whether nicotine or other constituents are the sole cause for the observed effects of ENDS use. Because this is a single-case study, certain limitations do exist. Alcohol, tobacco, and other drug use as well as folate consumption was assessed exclusively by self-report and could have been subject to certain demand characteristics. Frequency of ENDS use, the strength of e-liquid used in the ENDS product, and folate consumption assessed using the Block DFE screener may have been inaccurately estimated. Due to the study methodology, the risk of self-report bias does exist for this study. Furthermore, information about the generation of ENDS product used, flavoring, or variability of either use was not gathered from this study. The perceived impact ENDS use has on serum folate status cannot be generalized from a single-case study. Further research, including observational studies with larger populations, is needed to determine whether the finding can be replicated.
